# pSuc-FFSEA: Predicting Lysine Succinylation Sites in Proteins Based on Feature Fusion and Stacking Ensemble Algorithm

**DOI:** 10.3389/fcell.2022.894874

**Published:** 2022-05-24

**Authors:** Jianhua Jia, Genqiang Wu, Wangren Qiu

**Affiliations:** Computer Department, Jingdezhen Ceramic University, Jingdezhen, China

**Keywords:** post-translational modifications, succinylation, feature fusion, stacking ensemble, broad learning system, bayesian optimization

## Abstract

Being a new type of widespread protein post-translational modifications discovered in recent years, succinylation plays a key role in protein conformational regulation and cellular function regulation. Numerous studies have shown that succinylation modifications are closely associated with the development of many diseases. In order to gain insight into the mechanism of succinylation, it is vital to identify lysine succinylation sites. However, experimental identification of succinylation sites is time-consuming and laborious, and traditional identification tools are unable to meet the rapid growth of datasets. Therefore, to solve this problem, we developed a new predictor named pSuc-FFSEA, which can predict succinylation sites in protein sequences by feature fusion and stacking ensemble algorithm. Specifically, the sequence information and physicochemical properties were first extracted using EBGW, One-Hot, continuous bag-of-words, chaos game representation, and AAF_DWT. Following that, feature selection was performed, which applied LASSO to select the optimal subset of features for the classifier, and then, stacking ensemble classifier was designed using two-layer stacking ensemble, selecting three classifiers, SVM, broad learning system and LightGBM classifier, as the base classifiers of the first layer, using logistic regression classifier as the meta classifier of the second layer. In order to further improve the model prediction accuracy and reduce the computational effort, bayesian optimization algorithm and grid search algorithm were utilized to optimize the hyperparameters of the classifier. Finally, the results of rigorous 10-fold cross-validation indicated our predictor showed excellent robustness and performed better than the previous prediction tools, which achieved an average prediction accuracy of 0.7773 ± 0.0120. Besides, for the convenience of the most experimental scientists, a user-friendly and comprehensive web-server for pSuc-FFSEA has been established at https://bio.cangmang.xyz/pSuc-FFSEA, by which one can easily obtain the expected data and results without going through the complicated mathematics.

## 1 Introduction

Protein post-translational modifications (PTMs) is an important mechanism for regulating the function of protein, which plays an irreplaceable role in biological processes and signal pathways, and reversibly determines cell dynamics and plasticity (Xue et al., 2011). In recent years, lysine succinylation has been found to be a novel type of PTMs defined as transfer of a succinyl group ( $-\text{CO}-{\text{CH}}_{2}-{\text{CH}}_{2}-\text{CO}-)\;$ to a lysine residue of protein molecule, which has attracted much attention from many researchers in China and abroad (Peng et al., 2011). Succinylation is a widely conserved post-translational modification of proteins present in prokaryotic and eukaryotic cells that orchestrate various biological processes such as gene expression (Weinert et al., 2013). It will result in more substantial changes in the chemical structure of lysine than the methylation and acetylation that occur on lysine (Li et al., 2014). Meanwhile, dysregulation of lysine succinylation is closely associated with many human diseases, including inflammation, cancer, tuberculosis, neurodegenerative diseases, allergic dermatitis, etc (Ao et al., 2021). In 2013, Park et al. also revealed the potential impacts of succinylation on mitochondrial metabolism-related enzymes and demonstrated the important role of succinylation in the regulation of metabolism (Park et al., 2013).

Many studies have also confirmed the prevalence of protein succinylation modifications in prokaryotes and eukaryotes. Succinylation was found to occur at the active site of high serine transfer succinylases. Succinylation may have effect on the central nervous system in *E. coli* (Kawai et al., 2006). In mycobacterium tuberculosis, succinylated proteins are involved in many processes, including transcription, translation, stress response, protein interactions, etc (Xie et al., 2015). In 2015, Yang et al. also indicated that lysine succinylation can dynamically regulate enzymes in carbon metabolism in both bacteria and human cells and play major roles in regulating the process of the metabolism in mycobacterium tuberculosis (Yang et al., 2015). Therefore, identification of succinylation sites is helpful for further understanding the cellular functions of proteins and the implementation of relevant pathological reseach, and provides some valuable clues for biomedical research and drug development.

Currently, some traditional experimental methods have been proposed to identify lysine succinylation sites such as high performance liquid chromatography assays, mass spectrometry and liquid chromatography-mass spectrometry (Lind et al., 2002). Although the traditional experimental methods have high accuracy in identifying succinylation sites, it requires a lot of manpower and time cost, and there are also some problems such as high false positives. Therefore, it is urgent to propose a new method to solve the shortcomings of traditional experimental methods.

In fact, during the last decade or so, a host of researchers have continued to propose effective methods and developed many rapid bioinformatics tools to identify succinylation sites in proteins in order to compensate for the shortcomings of traditional techniques (Chen et al., 2019; Li et al., 2020), such as traditional maching learning, deep learning, broad learning system (BLS) and so on. The traditional machine learning has also become a common method for identifying succinylation sites. In 2015, Xu et al. developed a SVM-based predictor called iSuc-PseAAC, but which did not take into account the distribution of the dataset (Xu et al., 2015). In 2016, Jia et al. proposed two prediction models: pSuc-Lys (Jia et al., 2016b) and iSuc-PseOpt (Jia et al., 2016a), however, some important sequence information is missing in these classifiers, in addition, iSuc-PseOpt merged sequence coupling effects onto the pseudo-components and optimized the imbalance dataset, but the performance of classifier is highly data-dependant. With using the latest datasets of a number of novel succinylation sites from the latest high-throughput proteomic assays, Hasan et al. constructed a predictor named SuccinSite in 2016, which introduced amino acid pattern and properties into random forest (RF) classifier to predict the lysine succinylation sites (Hasan et al., 2016). In 2017, Dehzangi et al. developed a predictor called PSSM-Suc which used a position-specific scoring matrix (PSSM) introduced into the binary model for feature extraction and used amino acid evolutionary information to predict succinylation (Dehzangi et al., 2017). Thereafter, Dehzangi et al. also proposed a predictor named SSEvol-Suc (Dehzangi et al., 2018) in 2018, which primarily integrated secondary structure and PSSM via atlas bipartite mapping into an AdaBoost classifier for predicting succinylation sites, which achieved significant improvements over the iSuc-PseAAC, iSuc-PseOpt, SuccinSite, and pSuc-Lys predictors. In the same year, Hasan et al. structured a predictor named GPSuc through using an logistic regression (LR) to combine the outputs of distinct RF scores. In 2020, IFS-LightGBM used a combination of the LightGBM feature selection method and the incremental feature felection (IFS) method to select the optimal subset of features that extracted multiple types of feature information (Zhang et al., 2020). In 2021, Ge et al. proposed a method named SuccSPred to predict succinylation sites by fusing feature, ranking method and parsimonious bayes to identify succinylation sites (Ge et al., 2021). Clearly, considerable progress has been made in the prediction of lysine succinylation sites based on the traditional machine learning.

As time goes on and technology advances, deep learning and broad learning system have been being applied to bioinformatics. In 2020, Ning et al. created HybridSucc, which integrated ten types of information features, introduced deep neural network (DNN) and penalized logistic regression (PLR) algorithms into the hybrid learning architecture to build the model (Ning et al., 2020). In the same year, Thapa et al. developed DeepSuccinylSite which used deep learning methods to identify succinylation sites through embedding and a thermal encoding (Thapa et al., 2020). In 2021, Huang et al. combined a long short-term memory (LSTM) and convolutional neural network (CNN) into a deep learning method for predicting lysine succinylation sites (Huang et al., 2021). Although existing deep learning-based methods can effectively predict the succinylation sites, most of them suffer from the time-consuming training process because of a number of hyperparameters and complicated structures. Therefore, after consulting the relevant literature, it is found that the BLS aims to offer an alternative way of learning in deep structure, and can lead to a promising performance in classification. The successful application of the BLS in predicting IncRNA-protein interactions (Fan and Zhang, 2019) makes it possible to use the BLS in this study, so after studying and researching deeply the BLS, the BLS has been applied to this study.

**FIGURE 1:**
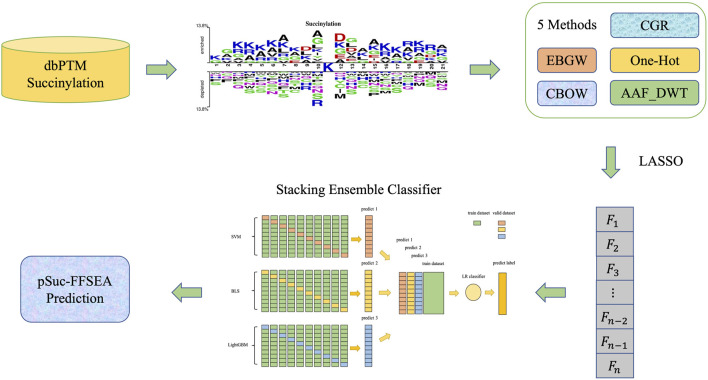
The scheme diagram for establishing pSuc-FFSEA model.

In order to improve the prediction performance of succinylation sites, the present study was initiated in an attempt to develop a new predictor based on feature fusion and stacking ensemble algorithm, which was proposed to identify lysine succinylation sites in protein sequences. The predictor is called “pSuc-FFSEA”, where “p" stands for “prediction”, “Suc” stands for “Succinylation sites”, “FF” stands for “Feature Fusion”, and “SEA” stands for “Stacking Ensemble Algorithm”. Since the use of flowcharts can show the intrinsic mechanism of model construction more visually, we drew diagrams to demonstrate the general framework of pSuc-FFSEA (Figure 1) with the following flow: First, based on protein sequences, features were extracted using multiple feature extraction methods including an encoding based on grouping weights (EBGW), one hot encoding (One-Hot), continuous bag-of-words (CBOW), chaos game representation (CGR), and amino acid factor features based on discrete wavelet transform (AAF_DWT). Multiple features of each type were feature selected using LASSO to eliminate the redundant information in the original feature vector. Then, the hyperparameters of SVM, LR and LightGBM classifier were optimized using Bayesian optimization algorithm, while the hyperparameters of broad learning system (BLS) were optimized using grid search algorithm. Finally, the succinylation prediction model has been constructed by comparing several other classifiers through ten-fold cross-validation on the dbPTM dataset and selecting the stacking ensemble classifier as the predictive classifier, which was designed using two-layer stacking ensemble, selecting three classifiers, SVM, BLS and LightGBM classifier, as the base classifiers of the first layer, taking into account the variability among the base classifiers of the first layer and reducing overfitting, LR classifier was used as the meta classifier of the second layer. This work not only provided a better understanding of the sequence characteristics of protein succinylation modifications, but also provided a more effective algorithmic idea for directly predicting succinylation sites in proteins. Besides, for the convenience of the most experimental scientists, a user-friendly and comprehensive web-server for pSuc-FFSEA has been established at https://bio.cangmang.xyz/pSuc-FFSEA, by which one can easily obtain the expected data and results without going through the complicated mathematics.

## 2 Materials and Methods

This study described a new predictor called pSuc-FFSEA that took into account five types of sequence feature extraction methods including EBGW, One-Hot, CBOW, CGR, and AAF_DWT to predict succinylated and non-succinylated sites.The following subsections detail the benchmark dataset used in this study and how features were extracted for each segment of amino acids corresponding to lysine residues, in addition to a discussion of the design of the stacking ensemble algorithm for succinylation sites prediction and performance evaluation metrics.

### 2.1 Benchmark Dataset

The benchmark dataset used in this study is derived from dbPTM (Huang et al., 2016; Huang et al., 2019) (https://awi.cuhk.edu.cn/dbPTM/) a protein lysine modification database that integrates published literature, public resources and a total of 41 biological databases related to PTMs. We obtained 2599 protein sequences from the dbPTM as our final training set, including 5049 experimentally validated lysine succinylation and 5526 non-succinylation sites. For convenience, we have placed the dataset on github, which is available at https://github.com/wugenqiang/pSuc-FFSEA/tree/main/dataset.

The protein sequence corresponding to lysine (K) was extracted from the dataset with a window size of 2*r* + 1, where one is the lysine (K) extracted as the central site of the protein sequence; *r* is equal to 10, which means that each of the upstream and downstream of the lysine is selected 10 amino acid residues; finally, a protein sequence of length 21 was obtained. In this case, the positive samples take the succinylation residues as the central sites.

For facilitating the description later, Chou’s peptide formulation was adopted (Chou, 2001). According to Chou’s method, all the protein sequences containing succinylation sites or not can be expressed as Eq. 1.

$$P_{\delta }\left(K\right)=H_{-\delta }H_{-\left(\delta -1\right)}\cdots H_{-2}H_{-1}KH_{+1}H_{+2}\cdots H_{+\left(\delta -1\right)}H_{+\delta }$$ (1)

where the center *K* represents lysine, the subscript δ represents an integer, the left half of *K* is the upstream amino acid residue, and the right half is the downstream amino acid residue, $H_{-\delta }$ represents the δth upstream amino acid residue counting from the center, and $H_{+\delta }$ represents the δth downstream amino acid residue counting from the center, so that $P_{\delta }\left(K\right)$ can divide all samples into two categories as defined in Eq. 2.

$$P_{\delta }\left(K\right)\in \{\begin{array}{cc} P_{\delta }^{+}\left(K\right), & if\;the\;center\;is\;a\;succinylation\;site\\ P_{\delta }^{-}\left(K\right), & otherwise \end{array}$$ (2)

Among them, $P_{\delta }^{+}\left(K\right)$ is expressed as a protein sequence with a lysine succinylated center; $P_{\delta }^{-}\left(K\right)$ is expressed as a protein sequence centered on a lysine unmodified succinylation.

As described in a review (Chou and Shen, 2007), if the predictor to be developed is a Jackknife test or a subsampling (or *K*-fold cross-validation) test, the benchmark dataset for the current study does not need to be split into separate testsets for further testing since the results obtained in this way are actually a combination of many different independent testsets. During the data preprocessing, it is not difficult to find that some peptide chain samples have some residues in the first or last part of the chain that are non-standard amino acids, such as “X", and the method introduced in Jia’s study (Jia et al., 2016b) can fill this part of residues by the mirroring image, as shown in formula 3 and 4.

(a) Mirror image for C terminus

$$H_{+\delta }H_{+\left(\delta -1\right)}\cdots H_{+2}H_{+1}\underset{K}{\Leftrightarrow }H_{+1}H_{+2}\cdots H_{+\left(\delta -1\right)}H_{+\delta }$$ (3)

(b) Mirror image for N terminus

$$H_{-\delta }H_{-\left(\delta -1\right)}\cdots H_{-2}H_{-1}\underset{K}{\Leftrightarrow }H_{-1}H_{-2}\cdots H_{-\left(\delta -1\right)}H_{-\delta }$$ (4)

According to Eqs 3 and 4, (a) and (b) are the mirror images of the carbon-terminus and nitrogen-terminus δ residues, respectively, on the left side of the symbol “⇔” in Eq. 3 and on the right side of the symbol “⇔” in Eq. 4; while the original protein sequence is on the other side, with the symbol "⇔" in the middle indicating the mirror image and *K* indicating the modification site.

### 2.2 Feature Extraction Methods

To build an effective prediction model, we encoded each protein sequence fragment as a numerical vector and inputed it as a feature into the model, which was the most critical step in proposing a classifier and integrating the architecture. Five feature extraction methods were used in this study including EBGW, One-Hot, CBOW, CGR, and AAF_DWT.

#### 2.2.1 EBGW

According to the idea of coarse-grained, if completely different things with the same characteristics, we can consider them as a whole. It is well known that it is the random combination of 20 amino acids with different properties that causes the diversity and specificity of protein structure and function (Zhang et al., 2006). Therefore, we decided to apply the physical and chemical properties of amino acids to capture the specific information between succinylated and non-succinylated sites.

Taking into account the hydrophobic, charged character and the coarse-grained idea, we divided the 20 amino acids into four groups as shown in Table 1.

**TABLE 1:** Classification of Amino acid residues.

Group	Amino Acid Residue	Characteristic
G1	A,F,G,I,L,M,P,V,W	neutral and non-polarity group
G2	C,N,Q,S,T,Y	neutral and polarity group
G3	D,E	acidic group
G4	H,K,R	basic group

These four groups of amino acids were further divided into three disjoint groups when the amino acid residues $P_{i}\left(i=1,2,\cdots ,n\right)$ appeared in protein sequence $P=\left(P_{1},P_{2},\cdots ,P_{n}\right)$ . Through this process, a protein sequence was converted into three binary sequences $S_{1},S_{2}$ and $S_{3}$ as defined in Eqs. 5-7, respectively.

$$S_{1}\left(P_{i}\right)=\{\begin{array}{cc} 1 & \;if\;P_{i}\in G_{1}\cup G_{2}\\ 0 & if\;Pi\in G_{3}\cup G_{4}\left(i=1,2,\cdots ,n\right) \end{array}$$ (5)

$$S_{2}\left(P_{i}\right)=\{\begin{array}{cc} 1 & \;if\;P_{i}\in G_{1}\cup G_{3}\\ 0 & if\;Pi\in G_{2}\cup G_{4}\left(i=1,2,\cdots ,n\right) \end{array}$$ (6)

$$S_{3}\left(P_{i}\right)=\{\begin{array}{cc} 1 & \;if\;P_{i}\in G_{1}\cup G_{4}\\ 0 & if\;Pi\in G_{2}\cup G_{3}\left(i=1,2,\cdots ,n\right) \end{array}$$ (7)

For convenience, we denoted $S\left(n\right)=s_{1},s_{2},\cdots ,s_{n}$ as any one of the three feature sequences defined above. The specific process is as follows.

Step 1: Define weights for feature sequences.

Suppose $S\left(n\right)=s_{1},s_{2},\cdots ,s_{n}$ be a sequence of features, and the weight of S(n) be defined as the number of times the number one appeares in S(n) .

Step 2: Standardized weights.

The standardized weight f(n) is defined as the frequency of occurrence of the number one in S(n) , that is $f\left(n\right)=\frac{w\left(n\right)}{n}$ , where w(n) is the weight of S(n) .

Step 3: Select the appropriate frequency value.

Assume K be a positive integer and *L* be the length of sequence, we can select K values in f(n) according to the following rules. Equal steps size is defined as $Q=\left(\left\lfloor \frac{L}{K}\right\rfloor \right)$ , ⌊·⌋ refers to a number down to the nearest integer. Then we can get P=[f(1×Q), (2×Q), ⋯, f(K×Q)] which we call as the EBGW string of feature sequence S(n) .

Thus, given a protein sequence, we can transform it into three feature sequences $S_{1},S_{2}$ and $S_{3}$ , which were performed from step 1 to step 3 in sequence in order. Finally, the protein sequences were encoded as 3 × *K*-dim feature vectors. Preliminary tests on the training dataset showed that *K* = 5 is the most appropriate number of points to be fetched. Therefore, a protein sequence of length 21 was converted into a 15-dim (5×3) feature vector.

#### 2.2.2 One-Hot

The most direct and basic features of protein sequences are the types and positions of amino acid residues, and One-Hot is the most intuitive way to express these two features; therefore, the One-Hot method has been maturely applied to the process of protein feature extraction. In order to extract features in the collected protein sequences for further complementation, the binary encoding method for extracting features is also applied to the tools in this paper. The 20 amino acid letters were listed in alphabetical sequence as: ACDEFGHIKLMNPQRSTVWY. The $i^{th}$ amino acid was expressed as one in the $i^{th}$ position and 0 in the other positions. For example, the binary representation of the amino acid A was written as 10000000000000000000, amino acid letter C was written as 01000000000000000000. In this regard, the protein sequence of length 21 was represented by 420-dim ( 21×20) vector.

#### 2.2.3 CBOW

The context of a word often has many words, and what we need is to predict the probability of occurrence of the missing word given multiple words. We want the bag-of-words model to handle this problem. Therefore, a new solution idea was proposed, which used the product of the average of the input context word vectors and the weights from the input layer to the hidden layer as input and the average of the context word vectors as output, and used the context in this way to predict the current word, which was the continuous bag-of-words model, or CBOW for short (Mikolov et al., 2013). Based on CBOW model, we constructed two different words embedded in wordbooks for training model and the corresponding feature vectors were generated using them (Qiu et al., 2021). The training process is as follows.

Step 1: Divide protein sequences into segments and create wordbooks.

**FIGURE 2:**
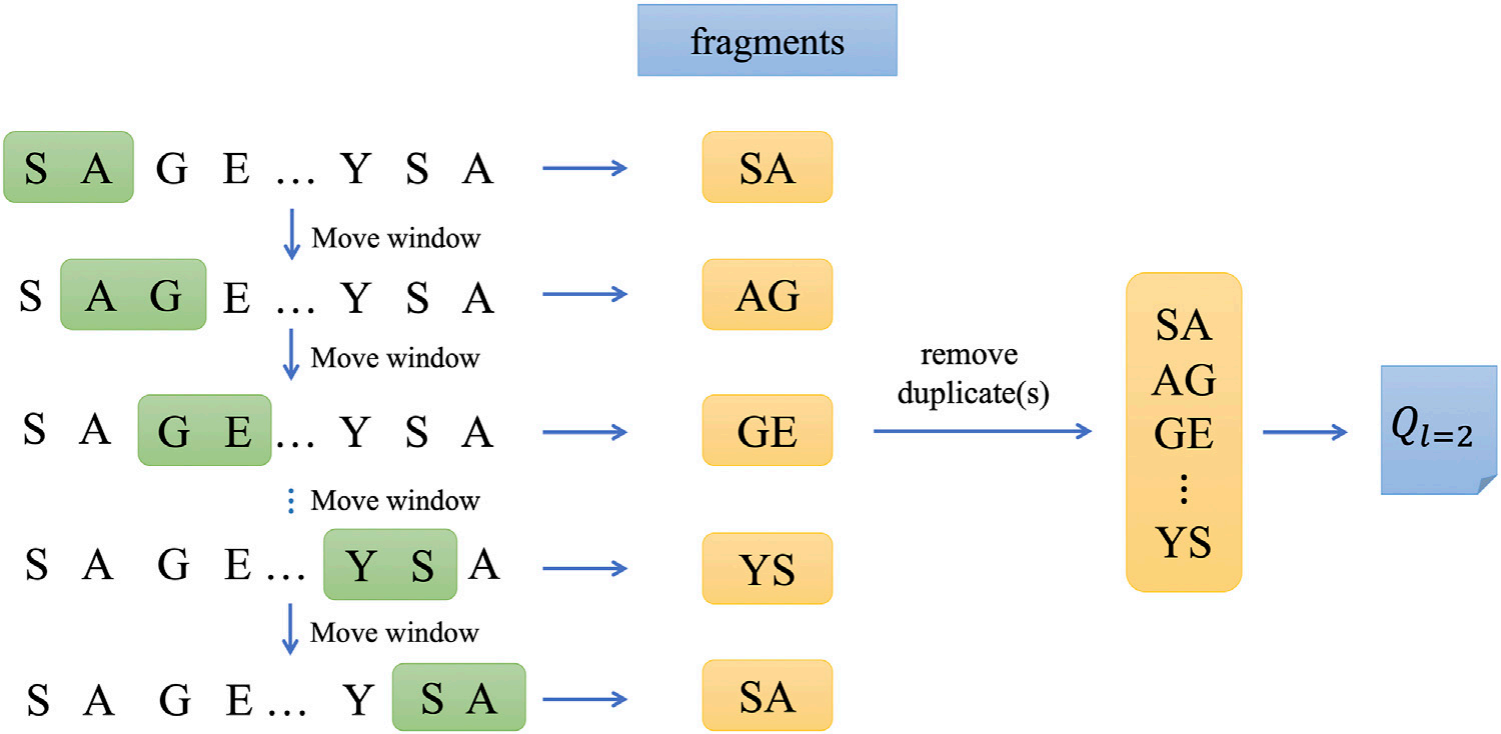
The process of splitting sequences and forming wordbook of $Q_{l=2}$ .

Two fragments are designed with length l of 2 and 3, respectively, and denoted as $Q_{l=2}$ and $Q_{l=3}$ , respectively, taking $Q_{l=2}$ as an example for illustration in this study. As shown in Figure 2, the original protein sequence is divided into words of length 2, i.e., setting the window size to two and moving the window in steps of 1. After all this work is done, the words split from each sequence will be collected, the duplicate items removed, and then a wordbook with word count *v* is generated.

Step 2: Train the CBOW model.

**FIGURE 3:**
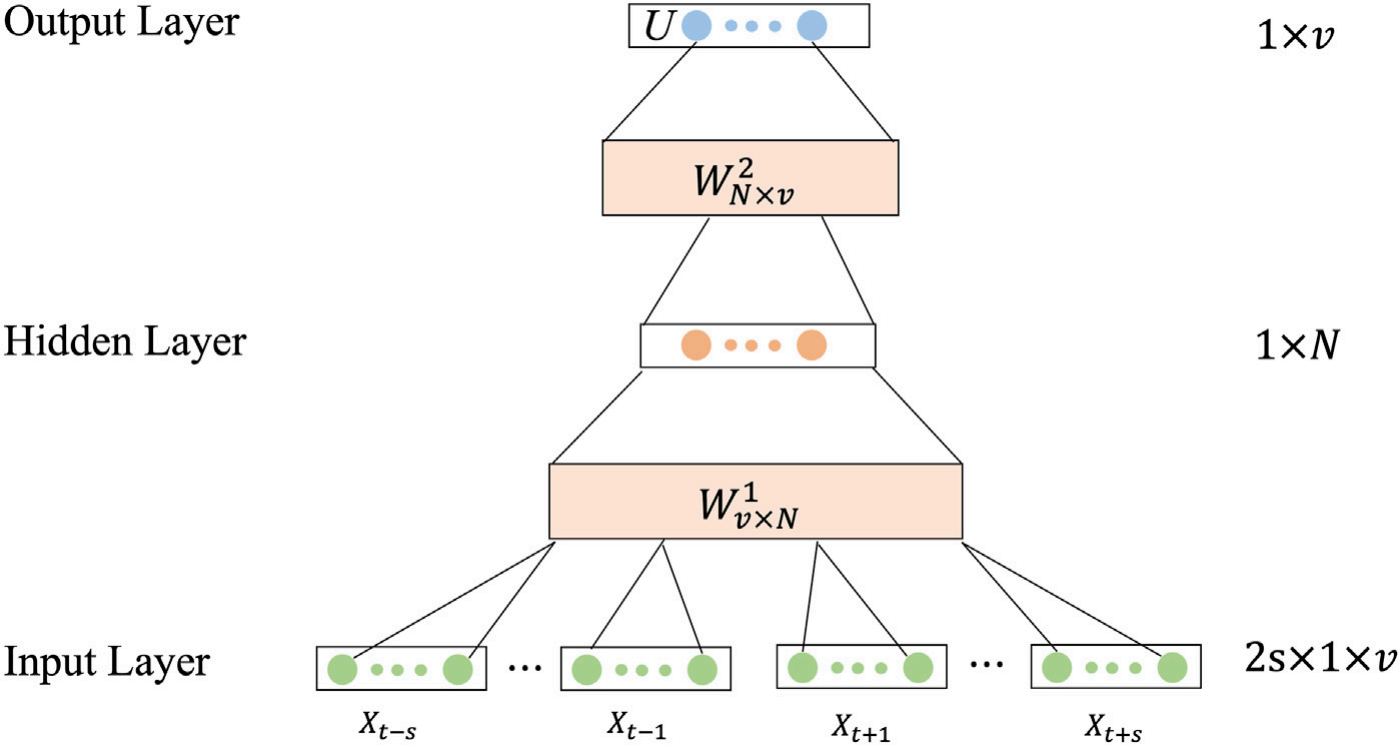
The structure of CBOW.

The CBOW model is applied to generate word vectors, and the target words are predicted according to the continuous words before and after the target words, and two word vector matrices are obtained by training the CBOW model.The structure of the CBOW model is shown in Figure 3.

Step 3: Feature extraction using CBOW model.

In this step, protein sequences are converted into feature vectors.

After performing the above steps, a protein sequence of length 21 was converted into a 200-dim (100×2) feature vector.

#### 2.2.4 CGR

In this study, we used CGR proposed by Jeffrey et al. to extract features (Joel, 1990). To achieve this, first, we transformed the protein sequences into nucleotide sequences according to Table 2, which was proposed by Deschavanne (Deschavanne and Tuffery, 2008). The advantage of using this code-switching method is the ability to maintain a balanced base composition for maximizing the differences between amino acids. Since the universal code that translates deoxyribose into amino acids is not unique, a wobble in the third base can lead to ambiguity in expression. Here, we assigned a unique codon to each amino acid as follows in Table 2.

**TABLE 2:** Reverse encoding for the amino acids used in this study.

A=GCT	C=TGC	D=GAC	E=GAG	F=TTC	G=GGT	H=CAC
I=ATT	K=AAG	L=CTA	M=ATG	N=AAC	P=CCA	Q=CAG
R=CGA	S=TCA	T=ACT	V=GTG	W=TGG	Y=TAC	

After the protein sequence was encoded using the unique codon as shown in Table 2, the corresponding nucleotide sequence was generated. Then the CGR generation operation was executed as follows: in the [0,1]×[0,1] square, the four vertices of the defined square corresponded to the four letters: A, C, G and T, as also detailed in Jia’s article (Jia et al., 2019). The CGR graph is obtained by the following steps.

Step 1: Place the starting point on the center in the square.
Step 2: Place the second point at the midpoint between the starting point and the vertex corresponding to the first nucleotide.
Step 3: Place the $i^{th}$ point between the ${\left(i-1\right)}^{th}$ point and the vertex corresponding to the $i^{th}$ nucleotide.
Step 4: Go to step 3 until the end of the nucleotide sequence is reached.

The above steps can be expressed using the formula as follows in Eq. 8.

$$CGR_{i}=\theta *\left(CGR_{i-1}+g_{i}\right)\;\;\;\;\;\;\;\;i=1,2,\cdots ,n_{G}$$ (8)

where $g_{i}$ denotes nucleotide coefficient, and when the nucleotides are A, C, G and T, the corresponding nucleotide coefficients are defined as (0, 0), (0, 1), (1, 0) and (1, 1). Considering the previous study, the parameter θ is set to 0.5. Also, we define $CGR_{0}=\left(0.5,\;0.5\right)$ and $i=1,\;2,\;\cdots ,\;n_{G}$ , $n_{G}$ is the length of a nucleotide sequence.

$$P_{\varphi }=\frac{1}{1+e^{-P_{\varphi }}}\;\;\;\;\left(\varphi =1,\;2,\;\cdots ,\;16\;\right)$$ (9)

After generating the CGR graph, the CGR square was divided into 4 × 4 = 16 sub-squares, each of which was of the same size. The number of points in each of the 16 sub-squares was calculated as a set of feature vectors $P=\left[P_{1},\;P_{2},\;\cdots ,\;P_{16}\right]$ . On this basis, the results were normalized using Eq. 9 to obtain a 16-dim feature vector.

#### 2.2.5 AAF_DWT

In this study, we considered to use the same ten physicochemical properties of amino acids as in the article (Jia et al., 2021) and the values of all physicochemical properties were extracted from AAindex (Kawashima et al., 2008), which can be obtained from https://github.com/wugenqiang/pSuc-FFSEA/blob/main/PP_Values.xlsx. The ten physicochemical properties of amino acids listed below: 1) consensus normalized hydrophobicity; 2) positive charge; 3) partition energy; 4) net charge; 5) conformational preference for all beta-strands; 6) conformational preference for antiparallel beta-strands; 7) mean polarity; 8) principal property value z3; 9) apparent partition energies calculated from Wertz-Scheraga index (10) weights from the IFH scale.

Suppose a protein sequence containing *L* amino acid residues is given and defined as Eq. 10.

$$P=R_{1}R_{2}\cdots R_{L}$$ (10)

$R_{1}$ denotes the first amino acid residue in the protein sequence. $R_{2}$ denotes the second, … … , $R_{L}$ denotes the last amino acid residue in protein sequence *P*. Protein sequence *P* under the $\xi^{th}$ physicochemical property can be expressed as Eq. 11.

$$P^{\left(\xi \right)}=\varphi_{1}^{\left(\xi \right)}\varphi_{2}^{\left(\xi \right)}\cdots \varphi_{L}^{\left(\xi \right)}\;\;\;\;\left(\xi =1,2,\cdots ,10\right)$$ (11)

where " ξ " denotes the $\xi^{th}$ physicochemical property and $\varphi_{i}^{\left(\xi \right)}$ denotes the value of the $\xi^{th}$ physicochemical property of the $i^{th}$ amino acid. Then the normalized transformation is performed as defined in Eq. 12.

$$\varphi_{i}^{\left(\xi \right)}=\frac{\varphi_{i}^{\left(\xi \right)}-M\left(\varphi_{i}^{\left(\xi \right)}\right)}{STD\left(\varphi_{i}^{\left(\xi \right)}\right)}\left(\xi =1,2,\cdots ,10;i=1,2,\cdots ,L\right)$$ (12)

The symbol “M" indicates the average of the 20 amino acid values and “STD” indicates the corresponding standard deviation. After standardization, the average value of the 20 amino acids is 0, which will remain unchanged if the same standard conversion procedure is performed again.

**FIGURE 4:**
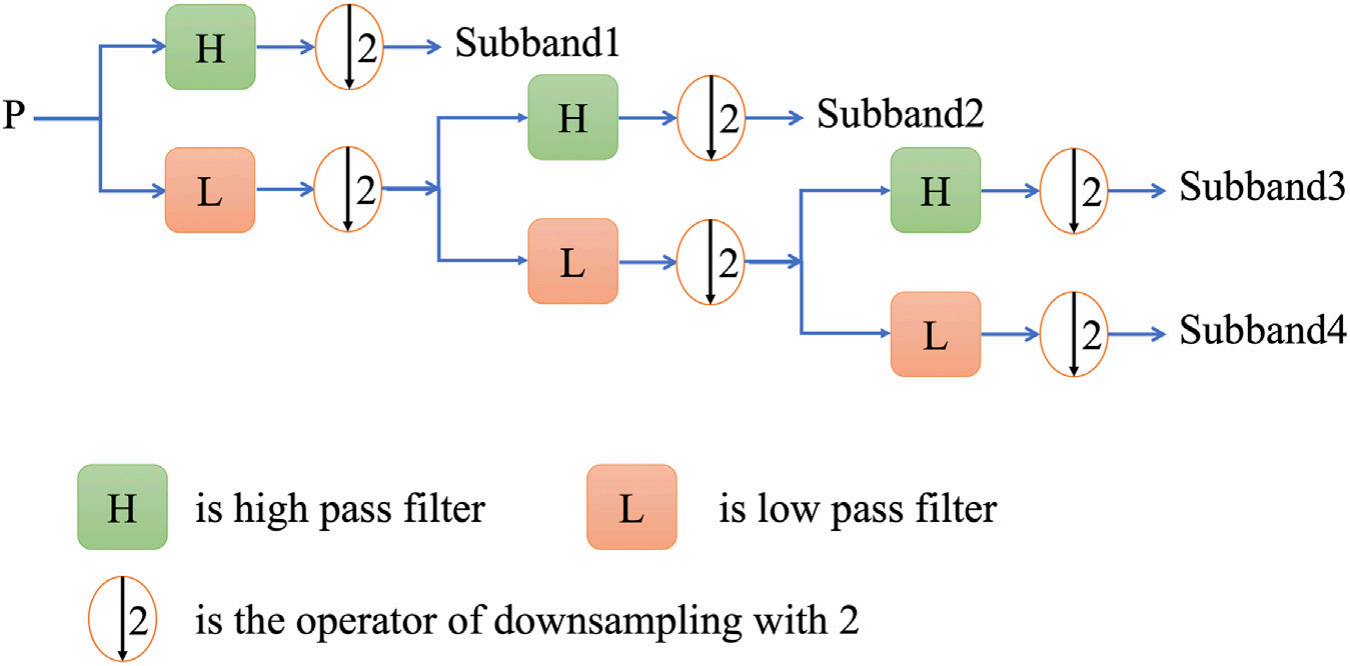
A schematic drawing to illustrate the procedure of multi-level DWT.

Wavelet transform is a new transform analysis method inherited and developes on the basis of fourier analysis (Mallat, 1989), which overcomes the previous shortcoming. As an ideal tool for signal time-frequency analysis and processing, its main feature is that it can highlight some important features. Discrete wavelet transform (DWT) is to discretize the scale and translation of the fundamental wavelet, which can convert the discrete time signal into discrete wavelet representation (Shensa, 1992). When applying the discrete wavelet transform to feature extraction, $P^{\left(\xi \right)}$ can be considered as a discrete time series, with the first amino acid residue corresponding to *t*=1, the second to *t*=2, and so on. The time series is then used as input to a high-pass filter and a low-pass filter, and the resulting coefficients can be approximated for both the high-frequency and low-frequency components of the signal. The digital implementation of DWT is shown in Figure 4.

In this study, the Harr wavelet was selected as wavelet basis function in the specific implementation process and λ=3 was chosen as the decomposition level of DWT to represent a protein sequence. After standardization, the protein sequence *P* applied DWT to obtain (3+1)=4 subbands, and each subband contained four coefficients, which are: 1) $\alpha_{i}$ : the maximum value of wavelet coefficients of the $i^{th}$ subband; 2) $\beta_{i}$ : the minimum value of wavelet coefficients of the $i^{th}$ subband; 3) $\gamma_{i}$ : the mean value of wavelet coefficients of the $i^{th}$ subband; 4) $\delta_{i}$ : the standard deviation of the $i^{th}$ subband wavelet coefficients (*i*=1,2,3,4). Accordingly, a new formula is defined as Eq. 13.

$$\psi_{j}=\{\begin{array}{cc} \alpha_{j} & if\;1\leq j\leq 4\\ \beta_{j} & if\;5\leq j\leq 8\\ \gamma_{j} & if\;9\leq j\leq 12\\ \delta_{j} & if\;13\leq j\leq 16 \end{array}$$ (13)

Therefore, after the wavelet transform with λ=3 , the peptide *P* can be re-expressed under the $\xi^{th}$ physicochemical property as Eq. 14.

$$P\prime^{\left(\xi \right)}=\psi_{1}^{\left(\xi \right)}\psi_{2}^{\left(\xi \right)}\cdots \psi_{16}^{\left(\xi \right)}\;\;\;\;\left(\xi =1,2,\cdots ,10\right)$$ (14)

Finally, the discrete wavelet transform was combined with the physicochemical properties of amino acids to obtain the final protein sequence features as defined in Eq. 15.

$$P=\left[P\prime^{\left(1\right)},\;P\prime^{\left(2\right)},\;\cdots ,P\prime^{\left(10\right)}\right]$$ (15)

After performing the above steps, a protein sequence of length 21 was converted into a 160-dim (16×10) feature vector.

To extract more feature information from protein sequences, We fused five feature extraction methods to obtain a total of 811-dim feature vectors from each protein sequence.

### 2.3 Feature Selection Method

High dimensional feature sets usually contain noise and redundant features that are unfavorable to the prediction performance of the model. Before building the model, it is necessary to select the optimal subset of features through feature selection to reduce the dimensionality of the feature space and further reduce the risk of overfitting. Meanwhile, removing irrelevant features before training can achieve better the generalization performance and prediction ability of the model. In this paper, LASSO (least absolute shrinkage and selection operator) was used for feature selection to form the optimal feature subset of the independent variables to improve the prediction performance of the model (Wang, 2010).

This method has been successfully applied to predict protein ubiquitination sites (Xca et al., 2019), tumor classification (Kang et al., 2018), and drug-target interactions prediction (Han et al., 2019). The basic idea of LASSO is to introduce *L*1 norm regularization from minimizing residuals sum of squares. The LASSO sparse representation coefficient *w* can be described as shown in Eq. 16.

$$J\left(w\right)=\underset{w}{\min}\sum_{i=1}^{k}{\left(y_{i}-w^{T}x_{i}\right)}^{2}+\lambda \|w{\|}_{1}$$ (16)

where $x_{i}$ represents the feature of each protein sequence and $y_{i}$ represents the label of each protein sequence. The regularization parameter λ controls the degree of punishment of the sparse coefficient estimation, $w_{1}$ is a *L*1 norm. Eq. 16 is optimized using the coordinate gradient descent method.

### 2.4 Prediction Model Construction

#### 2.4.1 Base Classifiers and Meta Classifier

Since the selection of classifiers plays a crucial role in constructing an effective prediction model for succinylation sites, after testing, SVM, BLS and LightGBM were finally selected as the base classifiers used in this study, and LR was selected as the meta classifier used in the study, and then the stacking ensemble classifier was constructed on the basis of these four base classifiers.

SVM(Ju and Gu, 2016) is such an algorithm that strives to minimize the structural risk, which shows many unique advantages in solving small sample, nonlinear and high-dimensional pattern recognition.

LightGBM(Meng, 2018) is also an ensemble decision tree based model that uses gradient boosting techniques, it has faster training speed, higher efficiency, lower memory consumption and better accuracy (Zhou et al., 2020).

BLS (Chen and Liu, 2018) is a powerful algorithm for offering an alternative way of learning in deep structure, which is designed based on the idea of taking the mapping feature as the input of RVFLNN. The most important part of BLS is mapping from input value to characteristic value and it can update the modeling system step by step without retaining from the scratch.

LR is often appiled to find the relationship between the predictors and binary responses. The main idea of LR classification is to establish a regression formula for the classification boundary according to the existing data, and to classify it.

#### 2.4.2 Stacking Ensemble Classifier

Stacking ensemble algorithm is an ensemble machine learning algorithm, which uses meta-learning algorithms to learn how best to combine predictions from two or more base-learning algorithms. The advantage of the stacking ensemble algorithm is that it can take advantage of the ability of a series of well-performing models to classify tasks and make better predictions than any of the models in the ensemble algorithm.

**FIGURE 5:**
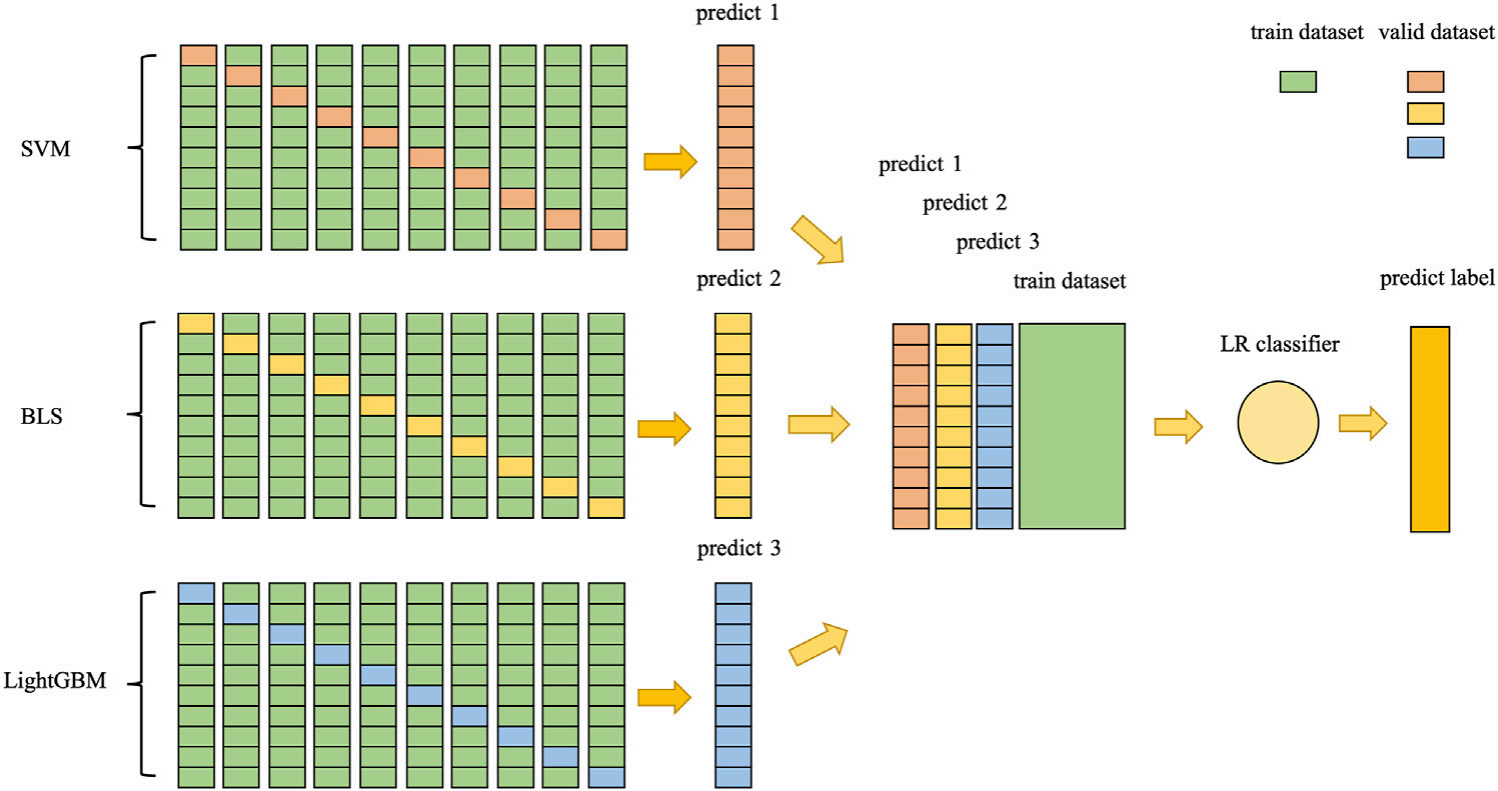
The framework of stacking ensemble classifier.

In general, the same predictive task will have different prediction results in different classifiers, and ensemble learning can use multiple classifiers to approximate the optimal target function. In this study, for the differences of different classifiers, stacking ensemble classifier was designed using two-layer stacking ensemble, selecting three classifiers, SVM, BLS and LightGBM classifier, as the base classifiers of the first layer, taking into account the variability among the base classifiers of the first layer, and also to reduce overfitting by integrating the output values of the first layer using LR classifier as the meta classifier of the second layer. Here, an optimization has been performed, i.e., the output values of the first layer was combined with the features extracted from the original dataset for stitching as the input to the second layer as represented in Figure 5. The stacking ensemble classifier can be represented by Eq. 17.

$$P\left(y=\pm 1|X\right)=\frac{1}{1+e^{-yw^{T}X}}\;\;\;\;\left(X=\left[x_{1},\;x_{2},\;x_{3},\;x_{original}\right]\right)$$ (17)

where X is a 305-dim vector spliced by the output values for SVM, BLS, LightGBM classifier and the features vectors extracted from the original dataset, w is the weight vector for the 305-dim vector.

### 2.5 Performance Evaluation

For any with *P* true positive samples and *N* true negative samples, there are four results of binary classification in the confusion matrix, namely true positive (TP), true negative (TN), false positive (FP) and false negative (FN). Then we can obtain five performance statistics as defined in Eq. 18.

$$\{\begin{array}{c} Sp=\frac{TN}{TN+FP}\\ Sn=\frac{TP}{TP+FN}\\ Acc=\frac{TP+TN}{TP+TN+FP+FN}\\ F1-Score=\frac{2\times TP}{2\times TP+FP+FN}\\ MCC=\frac{TP\times TN-FP\times FN}{\sqrt{\left(TP+FP\right)\times \left(TP+FN\right)\times \left(TN+FP\right)\times \left(TN+FN\right)}} \end{array}$$ (18)

To intuitively evaluate the predictive performance of our proposed succinylation predictor, we considered the use of five metrics: Sensitivity (Sn), Specificity (Sp), Accuracy (Acc), Mathews correlation coefficient (MCC), and F1-Score (Sokolova and Lapalme, 2009), Sn measures the proportion of positives correctly predicted, Sp measures the proportion of negatives correctly predicted, Acc measures the overall proportion of samples correctly predicted, and the F1-Score is a weighted summed average of accuracy and recall. MCC is considered to be one of the best measures (Boughorbel et al., 2017), even when the positive and negative distributions are very unbalanced. In general, -1 means that the prediction is completely wrong, 0 means that the categorical prediction is no better than the random prediction, and +1 means that the complete prediction is correct.

In addition, we used the ROC curve and the area under the ROC curve (AUC) to calculate the prediction performance of the predictor. To evaluate the performance of the proposed predictor using the previously mentioned performance metrics, the performance of the model was examined using 10-fold cross-validation. The purpose of 10-fold cross-validation is to verify the performance of the model, that is, to avoid the chance of the experiment, and to use the average of the results of 10 times to represent the overall performance of the model. The original dataset was randomly divided into 10 equal groups, and among the 10 groups, one group was selected as the testing set and the remaining nine groups were used as the training set, and then all performance metrics were calculated for each predictor. This is repeated 10 times by varying the training set and testing set from the 10 groups, and finally, the average of each performance metric was calculated for each predictor.

## 3 Results and Discussion

### 3.1 Sequence Analysis of Protein Lysine Succinylation Sites

**FIGURE 6:**
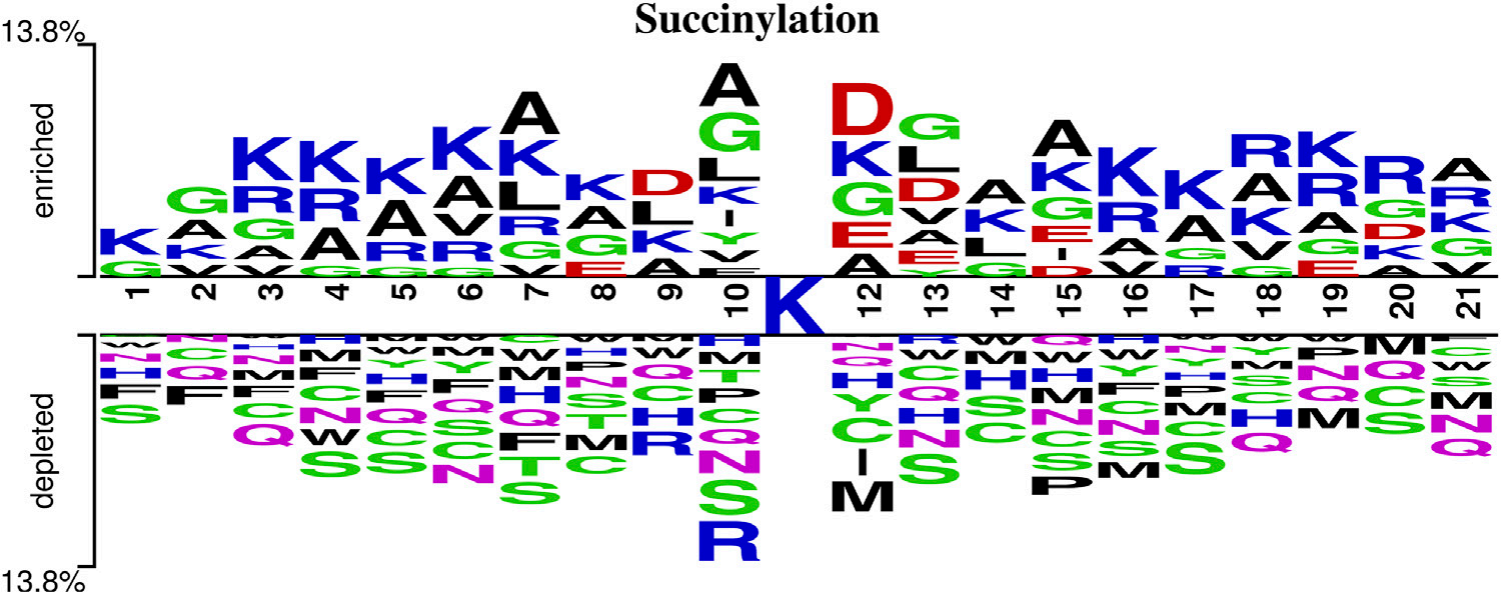
A two-sample logo of succinylation sites against non-succinylation sites. This logo is prepared using the web server http://www.twosamplelogo.org/and only residues significantly enriched and depleted surrounding succinylation sites (*t* test, *p*-value < 0.05) are shown.

To better analyze the differences between succinylation sites and non-succinylation sites in the protein sequences, this study used Two Sample Logo (Vacic et al., 2006) (http://www.twosamplelogo.org/) to analyze the protein sequences and investigated the frequency and position differences of 20 common amino acids near the succinylation sites and non-succinylation sites, as shown in Figure 6.

n this study, the sequence fragment length is 21, which included one central lysine, 10 upstream amino acids and 10 downstream amino acids. As shown in Figure 6, lysine (K), glycine (G), and alanine (A) appear more frequently around the succinylation site, and serine (S) appears more frequently around the non-succinylation site. Therefore, we concluded that the frequencies and positions of amino acid residues around the lysine succinylation site and the non-succinylation site are clearly different.

### 3.2 Effectiveness Analysis of Feature Extraction Methods

The information obtained by single feature extraction methods is often not comprehensive enough and the prediction results are not satisfactory. Multi-feature fusion can utilize different types of features to improve the prediction performance of the model. In this paper, the protein sequences were encoded using EBGW, One-Hot, CBOW, CGR and AAF_DWT to obtain 15-dim, 420-dim, 200-dim, 16-dim and 160-dim feature vectors, respectively. These five types of feature vectors were fused to obtain the fused feature set named ALL, the feature vectors extracted by five single feature extraction methods and the fused feature vectors vwere input into the model constructed based on the stacking ensemble classifier proposed in this study and the prediction results of different feature extraction methods for Sn, Sp, Acc, and MCC are shown in Table 3.

**TABLE 3:** Performance comparison of different feature extraction methods on the training set according to ten-fold cross-validation based on the stacking ensemble classifier proposed in this study.

Methods	Sn	Sp	Acc	MCC
EBGW	0.5783 ± 0.0245	0.6533 ± 0.0267	0.6175 ± 0.0175	0.2324 ± 0.0350
One-Hot	0.7283 ± 0.0189	0.7354 ± 0.0184	0.7320 ± 0.0139	0.4635 ± 0.0278
CBOW	0.6817 ± 0.0435	0.7463 ± 0.0431	0.7155 ± 0.0164	0.4305 ± 0.0328
CGR	0.5621 ± 0.0334	0.6672 ± 0.0173	0.6170 ± 0.0109	0.2308 ± 0.0225
AAF_DWT	0.6160 ± 0.0377	0.6638 ± 0.0425	0.6409 ± 0.0132	0.2808 ± 0.0263
ALL	0.7491 ± 0.0158	0.7651 ± 0.0208	0.7574 ± 0.0150	0.5142 ± 0.0298

In Table 3, it can be seen that different feature extraction methods correspond to different prediction results. among the five single feature extraction methods, Sn, Acc, and MCC of One-Hot reach the highest values, 0.7283 ± 0.0189, 0.7320 ± 0.0139, and 0.4635 ± 0.0278, respectively. Sn, Acc, and MCC of CGR are the lowest, 0.5621 ± 0.0334, 0.6170 ± 0.0109, and 0.2308 ± 0.0225, respectively. After fusing these five features, Sn, Sp, Acc and MCC were 0.7491 ± 0.0158, 0.7651 ± 0.0208, 0.7574 ± 0.0150 and 0.5142 ± 0.0298, respectively. Compared with the single feature, the Sn, Sp, Acc and MCC of the fused feature method are increased by at least 2.1%, 1.9%, 2.5% and 5.1%, respectively. The results indicate that multiple feature fusion can improve the prediction accuracy of various indicators.

**FIGURE 7:**
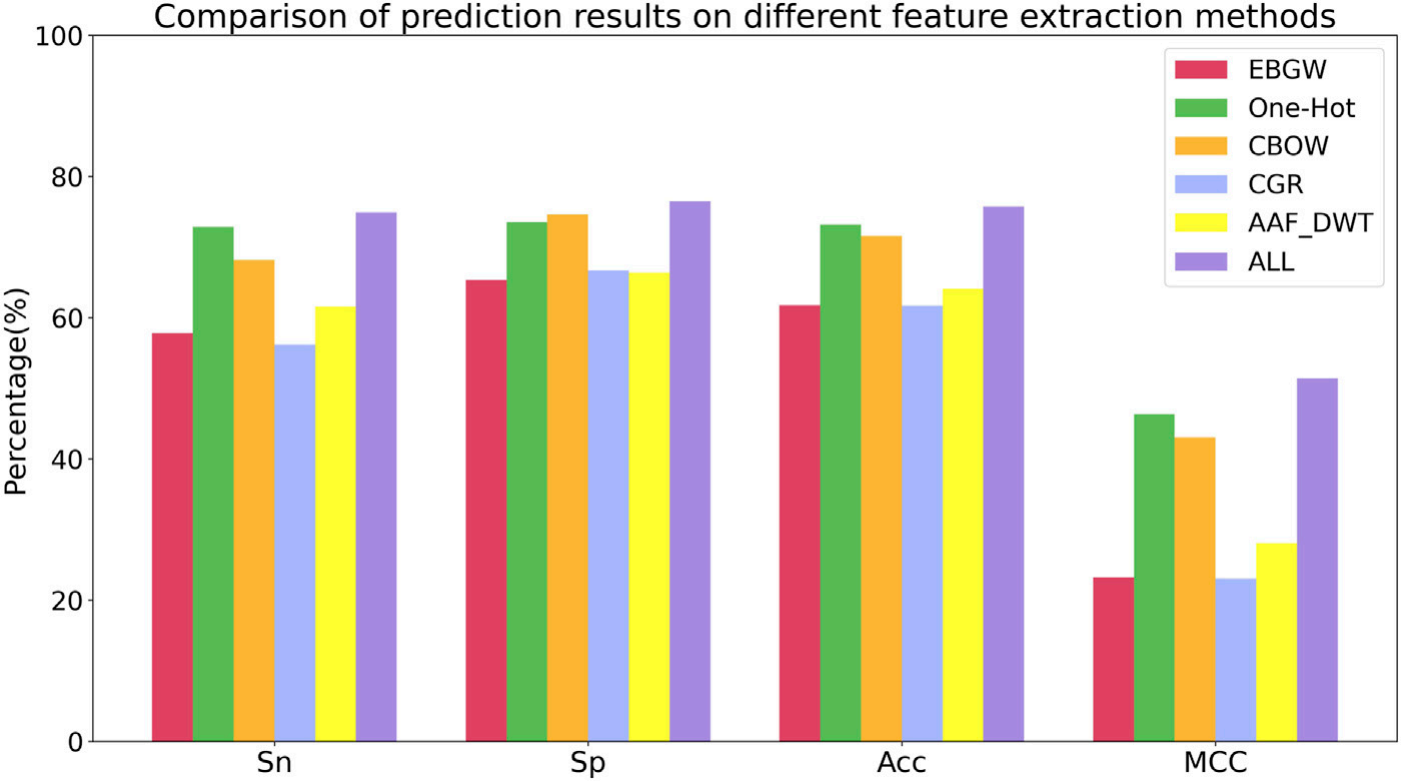
Comparison of prediction results on different feature extraction methods.

To better analyze the effects of different feature extraction methods on the prediction of succinylation sites, Figure 7 shows the histograms of Sn, Sp, Acc, and MCC for the six feature extraction methods.

In Figure 7, we can see that the six feature extraction methods have different effects on different evaluation indexes. Comparing the six feature extraction methods, the Sn, Sp, Acc and MCC of multi-feature fusion have the highest proportion, and it is shown that multi-feature fusion can make the information more comprehensive and improve the prediction ability of the model. Therefore, we used the multi-feature fusion method to extract protein sequence features for lysine succinylation sites prediction.

### 3.3 Effectiveness Analysis of LASSO

Multi-feature fusion extracts protein sequences and physicochemical information, but it generates redundant and noisy information, which will affect the prediction effect of the model, so feature selection is necessary to retain the important features for classification and further improve the computational efficiency of the model. In this study, we applied the method of LASSO to select the effective features from the 811-dim features of the fused feature dataset and obtained a subset of 302-dim features, which were input into the stacking ensemble classifier for classification. As shown in Table 4 **,** Sn, Sp, Acc, and MCC of the feature subset obtained by the method of LASSO have been improved by 1.1%, 2.75%, 2%, and 4%, respectively, and the results illustrate that the feature subset obtained by LASSO with dimensionality reduction can improve the classification ability of stacking ensemble classifier.

**TABLE 4:** Effectiveness analysis of LASSO on the training set according to ten-fold cross-validation.

Methods	Sn	Sp	Acc	MCC
no-LASSO	0.7491 ± 0.0158	0.7651 ± 0.0208	0.7574 ± 0.0150	0.5142 ± 0.0298
LASSO	0.7606 ± 0.0290	0.7926 ± 0.0234	0.7773 ± 0.0120	0.5541 ± 0.0243

### 3.4 Effectiveness Analysis of Classifiers

To evaluate the effectiveness of the stacking ensemble classifier to predict succinylation sites, four commonly-used classifiers including LR, SVM, LightGBM, and BLS were selected to predict lysine succinylation sites in this paper. These classifiers were compared with the stacking ensemble classifier proposed in this study to show which classifier had better performance.

In statistical prediction, the following three cross-validation methods were commonly used to derive predictor metrics values: independent dataset test, subsampling (or *K*-fold cross-validation) test, and Jackknife testing. And, of these three tests, the Jackknife test is considered the least arbitrary, always producing unique results for a given benchmark dataset, as described in Ref.(Chou, 2011). and proven by the equation, and ultimately, Jackknife has become widely accepted and increasingly used by researchers to test the performance of predictors. However, in order to reduce the computation time and to evaluate the prediction performance more fairly, like most researchers, we used 10-fold cross-validation to test the effectiveness of the method in this paper.

In order to make these classifiers have better prediction performance, we optimized the hyperparameters of these classifiers respectively. The hyperparameters of LR, SVM and LightGBM classifier were optimized using Bayesian optimization algorithm, while the hyperparameters of BLS were optimized using grid search algorithm. We found that the hyperparameters of LR is the best by default. The adjusted hyperparameters of SVM are as follows: kernel='linear’, C=1, and gamma=1. The adjusted hyperparameters of LightGBM are as follows: learning_rate=0.27, max_depth=40, num_leaves=51, and n_estimators=587. The adjusted hyperparameters of BLS are as follows: s=0.9, c=2**(-30), N1=3, N2=100, and N3=100.

**FIGURE 8:**
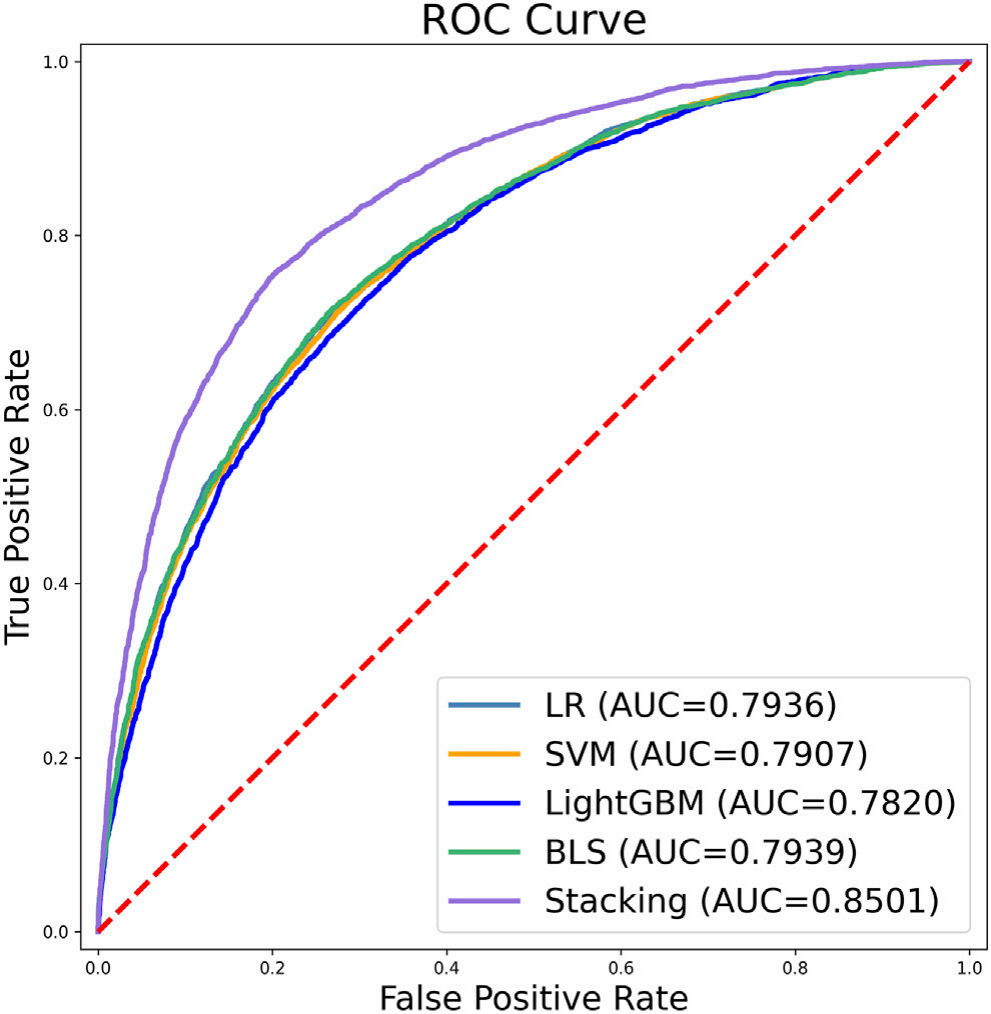
Receiver operating characteristics (ROC) curves for the five classifiers according to 10-fold cross-validation. The value of AUC represents the area under the ROC curve.

The results of the 10-fold cross-validation are summarized in Table 5 and Figure 8, where the stacking ensemble classifier predicts the best results in Table 5.

**TABLE 5:** Performance comparison of different classification algorithms on the training set according to ten-fold cross-validation.

Algorithm	Sn	Sp	Acc	MCC
LR	0.7017 ± 0.0258	0.7392 ± 0.0120	0.7213 ± 0.0133	0.4414 ± 0.0271
SVM	0.7029 ± 0.0227	0.7331 ± 0.0112	0.7187 ± 0.0128	0.4362 ± 0.0260
LightGBM	0.6924 ± 0.0213	0.7248 ± 0.0163	0.7093 ± 0.0112	0.4175 ± 0.0226
BLS	0.7043 ± 0.0268	0.7405 ± 0.0119	0.7232 ± 0.0130	0.4452 ± 0.0265
Stacking	0.7606 ± 0.0290	0.7926 ± 0.0234	0.7773 ± 0.0120	0.5541 ± 0.0243

Graphs are powerful tools for studying complex biological systems because they provide visual insight, and as demonstrated in a series of previous studies, receiver operating characteristic (ROC) graphs are used to show improvements in predictors in order to provide a visual comparison. the area under the ROC curve is called the AUC (area under the curve), and the larger the AUC value, the better the predictor. As shown in Figure 8.

Figure 8 shows that the stacking ensemble classifier has a higher accuracy ROC curve in the ten-fold cross-validation, and the area under the curve is 0.7936, 0.7907, 0.7820, 0.7939, 0.8501 for LR, SVM, LightGBM, BLS, and stacking ensemble classifier, respectively. The results show that the stacking ensemble classifier performs the best compared to the other four classifiers.

### 3.5 Comparison With the Existing Method

To prove the effectiveness of our predictor named pSuc-FFSEA, We performed a 10-fold cross-validation using the same training set to objectively compare pSuc-FFSEA with the existing methods, which are IFS-LightGBM(Zhang et al., 2020) and SuccSPred (Ge et al., 2021). IFS-LightGBM was constructed based on LightGBM classifier and the combination of the LightGBM feature selection method and the incremental feature selection method. SuccSPred was proposed to predict succinylation sites by fusing feature representation and ranking method. In order to improve the prediction effect, our predictor fused a variety of features and constructed the stacking ensemble classifier to predict succinylation sites.The performance comparison of the methods was shown in Table 6, it was found that pSuc-FFSEA has been significantly better than IFS-LightGBM in all metrics, Sn, Acc, MCC and F1-Score have been improved by 3.8%, 4.1%, 8.4% and 4.2%, respectively. For SuccSPred, Sn is 1.3% lower, meanwhile, ACC, MCC, F1-Score and AUC are 2.8%, 5.4%, 0.9% and 3.7% higher, respectively, and the results indicate that the proposed new predictor has better sensitivity, specificity, accuracy, F1-Score, and Mathews correlation coefficient.

**TABLE 6:** Performance comparison of pSuc-FFSEA with other existing methods.

Classifier	Sn	Sp	Acc	MCC	F1-score	AUC
IFS-LightGBM	0.7223	−	0.7360	0.4708	0.7232	−
SuccSPred	0.7731	−	0.7498	0.5001	0.7563	0.8132
pSuc-FFSEA	0.7606	0.7926	0.7773	0.5541	0.7651	0.8501

Therefore, we expect that pSuc-FFSEA may become a useful high-throughput tool in this important field, or at least complement existing methods.

### 3.6 Web Server and User Guide

**FIGURE 9:**
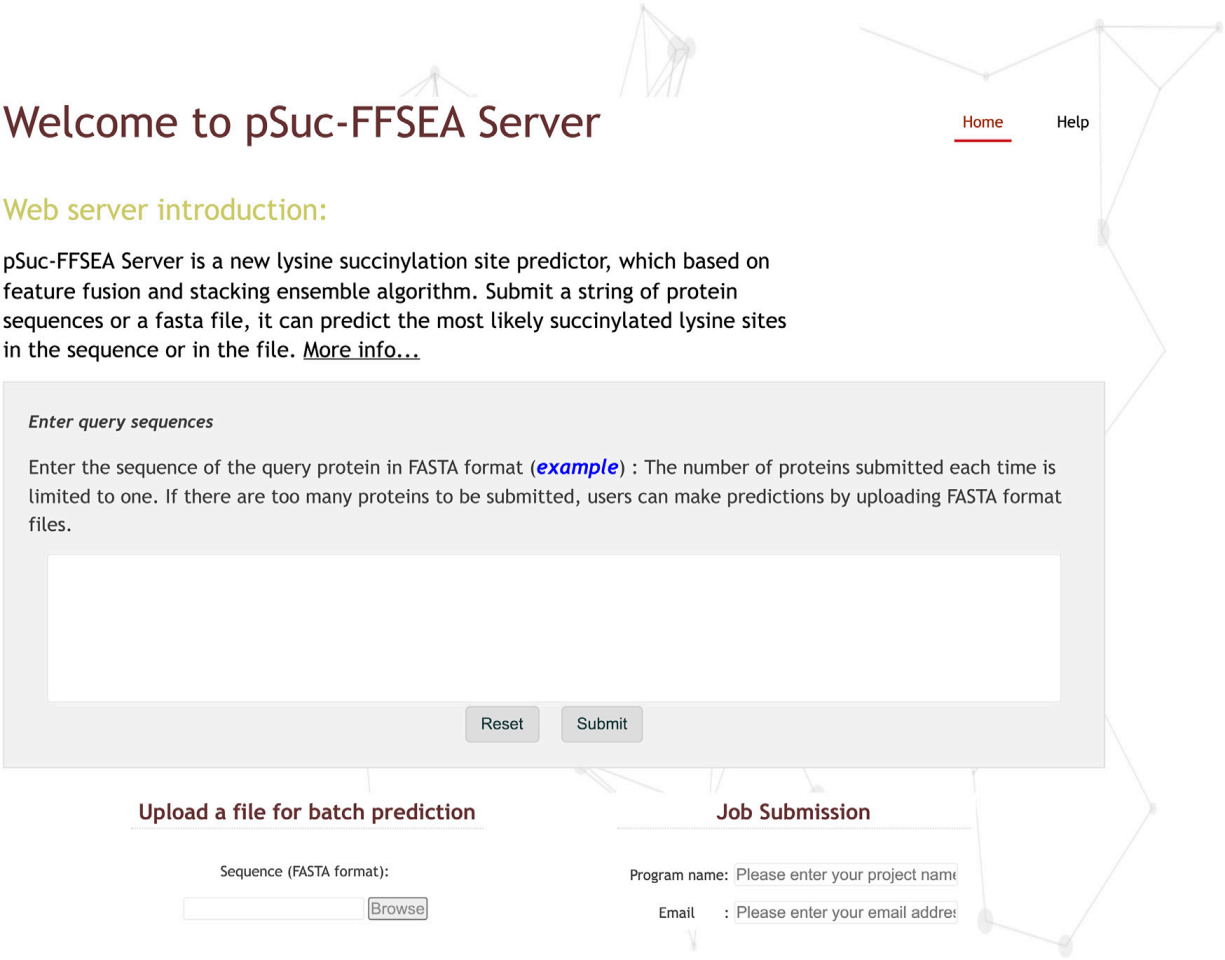
The screenshot to show the homepage of the pSuc-FFSEA web-server at https://bio.cangmang.xyz/pSuc-FFSEA.

In order to further enhance the practical application value of pSuc-FFSEA, based on all the above studies on lysine succinylation, an open online web server for pSuc-FFSEA has been established at https://bio.cangmang.xyz/pSuc-FFSEA. In addition, in order to maximize the convenience of most researchers, a guide to use is provided below:

Step 1: Use your browser to visit the website and you will see the homepage of pSuc-FFSEA as shown in Figure 9. Click on the “Help” or “More info..." button to see a brief introduction about the predictor.
Step 2: Enter or copy/paste a single protein sequence into the input box in the center of Figure 9. The input sequence should be in FASTA format. For an example of a sequence in FASTA format, click on the example button above the input box.
Step 3: After entering the protein sequence, click the “Submit” button to jump to the result page, where the lysine residues predicted to be succinylation sites are marked in red.
Step 4: The web server also provides a bulk protein prediction feature, which allows users to upload files via “Browse” button to upload a file and the file must be in FASTA format. And enter the project name and the email address to receive the prediction results, and finally click the “Submit” button, and the web server will send the prediction results to the user’s email address.

## 4 Conclusion

In this study, we constructed a novel and more effective predictor named pSuc-FFSEA based on feature fusion and stacking ensemble algorithm. Five feature extraction methods were fused, and these methods extracted sequence information and physical and chemical information features of peptide fragments based on EBGW, One-Hot, CBOW, CGR, and AAF_DWT, and then found the optimal feature representation using LASSO feature selection technique. Finally, the optimal model was built using the stacking ensemble classifier. This is also the first time to use BLS as a base classifier of stacking ensemble classifier to predict succinylation sites, pSuc-FFSEA can achieve relatively stable and high performance using the stacking ensemble classifier compared to existing predictors in this field.

For the convenience of most researchers, this study provided a web server and usage guide for pSuc-FFSEA, through which users can easily obtain the expected data and results without detailedly going through the complicated mathematics. The reason for including them in this paper is to make the new prediction method more sharable and testable, which will be beneficial to develop even more powerful methods for further predicting other PTM sites.

We anticipate that pSuc-FFSEA will be a very useful high-throughput tool, or at least a complementary tool to existing methods for predicting protein succinylation sites. In the future, the flexible application of feature extration methods and the optimization of classifiers will be the next step to explore in order to ease the difficulty of acquiring high-quality data. In this study, we also find the advantage of BLS, which may be even better than deep learning and traditional maching learning in various prediction problems. In the next work, we are going to apply deep learning to extract features and then use multiple BLS to build stacking ensemble classifier for the prediction of succinylation sites, which we believe will have more unexpected gains in the next experiments. With the development of proteomics research technology, the new methods will help to reveal the regulatory mechanism of lysine succinylation in normal physiological processes as well as pathological mechanisms, while succinylation has the potential to become the target of new drug action and provide new ideas for both biomedical research and drug development.

## Data Availability

Publicly available datasets were analyzed in this study. This data can be found here: https://github.com/wugenqiang/pSuc-FFSEA/tree/main/dataset.

## References

[B1] Ao C. Yu L. Zou Q. (2021). Prediction of Bio-Sequence Modifications and the Associations with Diseases. Brief. Funct. Genomics 20 (1), 1–18. 10.1093/bfgp/elaa023 33313647

[B2] Boughorbel S. Jarray F. El-Anbari M. (2017). Optimal Classifier for Imbalanced Data Using Matthews Correlation Coefficient Metric. PLoS One 12 (6), e0177678. 10.1371/journal.pone.0177678 28574989 PMC5456046

[B3] Chen C. L. P. Liu Z. (2018). Broad Learning System: An Effective and Efficient Incremental Learning System without the Need for Deep Architecture. IEEE Trans. Neural Netw. Learn. Syst. 29 (1), 10–24. 10.1109/TNNLS.2017.2716952 28742048

[B4] Chen Z. Liu X. Li F. Li C. Marquez-Lago T. Leier A. (2019). Large-scale Comparative Assessment of Computational Predictors for Lysine Post-translational Modification Sites. Brief. Bioinform 20(6)**,** 2267–2290. 10.1093/bib/bby089 30285084 PMC6954452

[B5] Chou K. C. (2001). Prediction of Protein Signal Sequences and Their Cleavage Sites. Proteins 42 (1), 136–139. 10.1002/1097-0134(20010101)42:1<136::aid-prot130>3.0.co;2-f 11093267

[B6] Chou K. C. Shen H. B. (2007). Recent Progress in Protein Subcellular Location Prediction. Anal. Biochem. 370 (1), 1–16. 10.1016/j.ab.2007.07.006 17698024

[B7] Chou K. C. (2011). Some Remarks on Protein Attribute Prediction and Pseudo Amino Acid Composition. J. Theor. Biol. 273 (1), 236–247. 10.1016/j.jtbi.2010.12.024 21168420 PMC7125570

[B8] Dehzangi A. Lopez Y. Lal S. P. Taherzadeh G. Michaelson J. Sattar A. (2017). PSSM-suc: Accurately Predicting Succinylation Using Position Specific Scoring Matrix into Bigram for Feature Extraction. J. Theor. Biol. 425, 97–102. 10.1016/j.jtbi.2017.05.005 28483566

[B9] Dehzangi A. Lopez Y. Lal S. P. Taherzadeh G. Sattar A. Tsunoda T. (2018). Improving Succinylation Prediction Accuracy by Incorporating the Secondary Structure via Helix, Strand and Coil, and Evolutionary Information from Profile Bigrams. PLoS One 13 (2), e0191900. 10.1371/journal.pone.0191900 29432431 PMC5809022

[B10] Deschavanne P. Tuffery P. (2008). Exploring an Alignment Free Approach for Protein Classification and Structural Class Prediction. Biochimie 90 (4), 615–625. 10.1016/j.biochi.2007.11.004 18067866

[B11] Fan X.-N. Zhang S.-W. (2019). LPI-BLS: Predicting lncRNA–Protein Interactions with a Broad Learning System-Based Stacked Ensemble Classifier. Neurocomputing 370, 88–93. 10.1016/j.neucom.2019.08.084

[B12] Ge R. Luo Y. Feng G. Jia G. Zhang H. Xu C. (2021). SuccSPred: Succinylation Sites Prediction Using Fused Feature Representation and Ranking Method. Bioinforma. Res. Applications,Lecture Notes Comput. Sci., 191–202. 10.1007/978-3-030-91415-8_17

[B13] Han S. Slab C. Jcab C. Xuan L. C. Qin M. Byab E. (2019). Predicting Drug-Target Interactions Using Lasso with Random Forest Based on Evolutionary Information and Chemical Structure. Genomics 111 (6), 1839–1852. 10.1016/j.ygeno.2018.12.007 30550813

[B14] Hasan M. M. Yang S. Zhou Y. Mollah M. N. (2016). SuccinSite: a Computational Tool for the Prediction of Protein Succinylation Sites by Exploiting the Amino Acid Patterns and Properties. Mol. Biosyst. 12 (3), 786–795. 10.1039/c5mb00853k 26739209

[B15] Huang G. Shen Q. Zhang G. Wang P. Yu Z. G. (2021). LSTMCNNsucc: A Bidirectional LSTM and CNN-Based Deep Learning Method for Predicting Lysine Succinylation Sites. Biomed. Res. Int. 2021, 9923112. 10.1155/2021/9923112 34159204 PMC8188601

[B16] Huang K. Y. Lee T. Y. Kao H. J. Ma C. T. Lee C. C. Lin T. H. (2019). dbPTM in 2019: Exploring Disease Association and Cross-Talk of Post-translational Modifications. Nucleic Acids Res. 47 (D1), D298–D308. 10.1093/nar/gky1074 30418626 PMC6323979

[B17] Huang K. Y. Su M. G. Kao H. J. Hsieh Y. C. Jhong J. H. Cheng K. H. (2016). dbPTM 2016: 10-year Anniversary of a Resource for Post-translational Modification of Proteins. Nucleic Acids Res. 44 (D1), D435–D446. 10.1093/nar/gkv1240 26578568 PMC4702878

[B18] Jeffrey H. J. (1990). Chaos Game Representation of Gene Structure. Nucl. Acids Res. 18 (8), 2163–2170. 10.1093/nar/18.8.2163 2336393 PMC330698

[B19] Jia J. Li X. Qiu W. Xiao X. Chou K. C. (2019). iPPI-PseAAC(CGR): Identify Protein-Protein Interactions by Incorporating Chaos Game Representation into PseAAC. J. Theor. Biol. 460, 195–203. 10.1016/j.jtbi.2018.10.021 30312687

[B20] Jia J. Liu Z. Xiao X. Liu B. Chou K. C. (2016a). iSuc-PseOpt: Identifying Lysine Succinylation Sites in Proteins by Incorporating Sequence-Coupling Effects into Pseudo Components and Optimizing Imbalanced Training Dataset. Anal. Biochem. 497, 48–56. 10.1016/j.ab.2015.12.009 26723495

[B21] Jia J. Liu Z. Xiao X. Liu B. Chou K. C. (2016b). pSuc-Lys: Predict Lysine Succinylation Sites in Proteins with PseAAC and Ensemble Random Forest Approach. J. Theor. Biol. 394, 223–230. 10.1016/j.jtbi.2016.01.020 26807806

[B22] Jia J. Shen Y. Qiu W. (2021). Identifying Lysine Succinylation Sites in Proteins by Broad Learning System and Optimizing Imbalanced Training Dataset via Randomly Labeling Samples. Wuhan Univ. J. Nat. Sci. 26 (01), 81–88. 10.19823/j.cnki.1007-1202.2021.0005

[B23] Ju Z. Gu H. (2016). Predicting Pupylation Sites in Prokaryotic Proteins Using Semi-supervised Self-Training Support Vector Machine Algorithm. Anal. Biochem. 507, 1–6. 10.1016/j.ab.2016.05.005 27197054

[B24] Kang C. Huo Y. Xin L. Tian B. Yu B. (2019). Feature Selection and Tumor Classification for Microarray Data Using Relaxed Lasso and Generalized Multi-Class Support Vector Machine. J. Theor. Biol. 463, 77–91. 10.1016/j.jtbi.2018.12.010 30537483

[B25] Kawai Y. Fujii H. Okada M. Tsuchie Y. Uchida K. Osawa T. (2006). Formation of Nepsilon-(succinyl)lysine *In Vivo*: a Novel Marker for Docosahexaenoic Acid-Derived Protein Modification. J. Lipid Res. 47 (7), 1386–1398. 10.1194/jlr.M600091-JLR200 16582421

[B26] Kawashima S. Pokarowski P. Pokarowska M. Kolinski A. Katayama T. Kanehisa M. (2008). AAindex: Amino Acid Index Database, Progress Report 2008. Nucleic Acids Res. 36, D202–D205. 10.1093/nar/gkm998 17998252 PMC2238890

[B27] Li F. Fan C. Marquez-Lago T. T. Leier A. Revote J. Jia C. (2020). PRISMOID: a Comprehensive 3D Structure Database for Post-translational Modifications and Mutations with Functional Impact. Brief. Bioinform 21 (3), 1069–1079. 10.1093/bib/bbz050 31161204 PMC7299293

[B28] Li X. Hu X. Wan Y. Xie G. Li X. Chen D. (2014). Systematic Identification of the Lysine Succinylation in the Protozoan Parasite Toxoplasma Gondii. J. Proteome Res. 13 (12), 6087–6095. 10.1021/pr500992r 25377623

[B29] Lind C. Gerdes R. Hamnell Y. Schuppe-Koistinen I. L?Wenhielm H. Holmgren A. (2002). Identification of S-Glutathionylated Cellular Proteins during Oxidative Stress and Constitutive Metabolism by Affinity Purification and Proteomic Analysis. Archives Biochem. Biophysics 406 (2), 229–240. 10.1016/s0003-9861(02)00468-x 12361711

[B30] Mallat S. G. (1989). A Theory for Multiresolution Signal Decomposition: the Wavelet Representation. IEEE Trans. Pattern Analysis Mach. Intell. 11 (4). 10.1109/34.192463

[B31] Meng Q. (2018). LightGBM: A Highly Efficient Gradient Boosting Decision Tree. NIPS'17: Proceedings of the 31st International Conference on Neural Information Processing Systems. 3149–3157. 10.5555/3294996.3295074

[B32] Mikolov T. Chen K. Corrado G. Dean J. (2013). Efficient Estimation of Word Representations in Vector Space. Comput. Sci. 10.48550/arXiv.1301.3781

[B33] Ning W. Xu H. Jiang P. Cheng H. Deng W. Guo Y. (2020). HybridSucc: A Hybrid-Learning Architecture for General and Species-specific Succinylation Site Prediction. Genomics Proteomics Bioinforma. 18 (2), 194–207. 10.1016/j.gpb.2019.11.010 PMC7647696 32861878

[B34] Park J. Chen Y. Tishkoff D. X. Peng C. Tan M. Dai L. (2013). SIRT5-mediated Lysine Desuccinylation Impacts Diverse Metabolic Pathways. Mol. Cell 50 (6), 919–930. 10.1016/j.molcel.2013.06.001 23806337 PMC3769971

[B35] Peng C. Lu Z. Xie Z. Cheng Z. Chen Y. Tan M. (2011). The First Identification of Lysine Malonylation Substrates and its Regulatory Enzyme. Mol. Cell Proteomics 10 (12), M111012658. 10.1074/mcp.M111.012658 PMC3237090 21908771

[B36] Qiu W. Lv Z. Xiao X. Shao S. Lin H. (2021). EMCBOW-GPCR: A Method for Identifying G-Protein Coupled Receptors Based on Word Embedding and Wordbooks. Comput. Struct. Biotechnol. J. 19, 4961–4969. 10.1016/j.csbj.2021.08.044 34527200 PMC8437786

[B37] Shensa M., J. (1992). The Discrete Wavelet Transform: Wedding the a Trous and Mallat Algorithms. Signal Process. IEEE Trans. 10.1109/78.157290

[B38] Sokolova M. Lapalme G. (2009). A Systematic Analysis of Performance Measures for Classification Tasks. Inf. Process. Manag. 45 (4), 427–437. 10.1016/j.ipm.2009.03.002

[B39] Thapa N. Chaudhari M. McManus S. Roy K. Newman R. H. Saigo H. (2020). DeepSuccinylSite: a Deep Learning Based Approach for Protein Succinylation Site Prediction. BMC Bioinforma. 21 (Suppl. 3), 63. 10.1186/s12859-020-3342-z PMC7178942 32321437

[B40] Vacic V. Iakoucheva L. M. Radivojac P. (2006). Two Sample Logo: a Graphical Representation of the Differences between Two Sets of Sequence Alignments. Bioinformatics 22 (12), 1536–1537. 10.1093/bioinformatics/btl151 16632492

[B41] Wang Z. (2010). A LASSO-type Approach to Variable Selection and Estimation for Censored Regression Model. Shanghai: chinese journal of applied probability and statistics.

[B42] Weinert B. T. Scholz C. Wagner S. A. Iesmantavicius V. Su D. Daniel J. A. (2013). Lysine Succinylation Is a Frequently Occurring Modification in Prokaryotes and Eukaryotes and Extensively Overlaps with Acetylation. Cell Rep. 4 (4), 842–851. 10.1016/j.celrep.2013.07.024 23954790

[B43] Xca B. Zya B. Byabc D. Mwa B. Bta B. Qin M. E. (2019). UbiSitePred: A Novel Method for Improving the Accuracy of Ubiquitination Sites Prediction by Using LASSO to Select the Optimal Chou's Pseudo Components - ScienceDirect. Chemom. Intelligent Laboratory Syst. 184, 28–43. 10.1016/j.chemolab.2018.11.012

[B44] Xie L. Liu W. Li Q. Chen S. Xu M. Huang Q. (2015). First Succinyl-Proteome Profiling of Extensively Drug-Resistant *Mycobacterium tuberculosis* Revealed Involvement of Succinylation in Cellular Physiology. J. Proteome Res. 14 (1), 107–119. 10.1021/pr500859a 25363132

[B45] Xu Y. Ding Y. X. Ding J. Lei Y. H. Wu L. Y. Deng N. Y. (2015). iSuc-PseAAC: Predicting Lysine Succinylation in Proteins by Incorporating Peptide Position-specific Propensity. Sci. Rep. 5, 10184. 10.1038/srep10184 26084794 PMC4471726

[B46] Xue Y. Liu Z. Cao J. Ren J. (2011). Computational Prediction of Post-Translational Modification Sites in Proteins. Syst. Comput. Biol. - Mol. Cell. Exp. Syst. 10.5772/18559

[B47] Yang M. Wang Y. Chen Y. Cheng Z. Gu J. Deng J. (2015). Succinylome Analysis Reveals the Involvement of Lysine Succinylation in Metabolism in Pathogenic *Mycobacterium tuberculosis* . Mol. Cell Proteomics 14 (4), 796–811. 10.1074/mcp.M114.045922 25605462 PMC4390261

[B48] Zhang L. Liu M. Qin X. Liu G. (2020). Succinylation Site Prediction Based on Protein Sequences Using the IFS-LightGBM (BO) Model. Comput. Math. Methods Med. 2020, 8858489. 10.1155/2020/8858489 33224267 PMC7673955

[B49] Zhang Z. H. Wang Z. H. Zhang Z. R. Wang Y. X. (2006). A Novel Method for Apoptosis Protein Subcellular Localization Prediction Combining Encoding Based on Grouped Weight and Support Vector Machine. FEBS Lett. 580 (26), 6169–6174. 10.1016/j.febslet.2006.10.017 17069811

[B50] Zhou K. Hu Y. Pan H. Kong L. Liu J. Huang Z. (2020). Fast Prediction of Reservoir Permeability Based on Embedded Feature Selection and LightGBM Using Direct Logging Data. Meas. Sci. Technol. 31 (4). 10.1088/1361-6501/ab4a45

